# Structure and Non-Structure of Centrosomal Proteins

**DOI:** 10.1371/journal.pone.0062633

**Published:** 2013-05-09

**Authors:** Helena G. Dos Santos, David Abia, Robert Janowski, Gulnahar Mortuza, Michela G. Bertero, Maïlys Boutin, Nayibe Guarín, Raúl Méndez-Giraldez, Alfonso Nuñez, Juan G. Pedrero, Pilar Redondo, María Sanz, Silvia Speroni, Florian Teichert, Marta Bruix, José M. Carazo, Cayetano Gonzalez, José Reina, José M. Valpuesta, Isabelle Vernos, Juan C. Zabala, Guillermo Montoya, Miquel Coll, Ugo Bastolla, Luis Serrano

**Affiliations:** 1 Centro de Biología Molecular Severo Ochoa (CBMSO), CSIC-UAM, Madrid, Spain; 2 Institut de Biologia Molecular de Barcelona (IBMB), Baldiri Reixac 10–12, Barcelona, Spain; 3 Institute for Research in Biomedicine (IRB-Barcelona) Baldiri Reixac 10–12, Barcelona, Spain; 4 Centro Nacional de Investigación Oncológica (CNIO), Madrid, Spain; 5 Centre for Genomic Regulation (CRG), Barcelona, Spain; 6 Instituto de Química Física Rocasolano (IQFR), CSIC, Madrid, Spain; 7 Centro Nacional de Biotecnología (CNB),-CSIC, Madrid, Spain; 8 Institucio Catalana de Recerca i Estudis Avançats (ICREA), Passeig Lluís Companys 23, Barcelona, Spain; 9 IFIMAV-Universidad de Cantabria, Santander, Spain; Università di Padova, Italy

## Abstract

Here we perform a large-scale study of the structural properties and the expression of proteins that constitute the human Centrosome. Centrosomal proteins tend to be larger than generic human proteins (control set), since their genes contain in average more exons (20.3 versus 14.6). They are rich in predicted disordered regions, which cover 57% of their length, compared to 39% in the general human proteome. They also contain several regions that are dually predicted to be disordered and coiled-coil at the same time: 55 proteins (15%) contain disordered and coiled-coil fragments that cover more than 20% of their length. Helices prevail over strands in regions homologous to known structures (47% predicted helical residues against 17% predicted as strands), and even more in the whole centrosomal proteome (52% against 7%), while for control human proteins 34.5% of the residues are predicted as helical and 12.8% are predicted as strands. This difference is mainly due to residues predicted as disordered and helical (30% in centrosomal and 9.4% in control proteins), which may correspond to alpha-helix forming molecular recognition features (α-MoRFs). We performed expression assays for 120 full-length centrosomal proteins and 72 domain constructs that we have predicted to be globular. These full-length proteins are often insoluble: Only 39 out of 120 expressed proteins (32%) and 19 out of 72 domains (26%) were soluble. We built or retrieved structural models for 277 out of 361 human proteins whose centrosomal localization has been experimentally verified. We could not find any suitable structural template with more than 20% sequence identity for 84 centrosomal proteins (23%), for which around 74% of the residues are predicted to be disordered or coiled-coils. The three-dimensional models that we built are available at http://ub.cbm.uam.es/centrosome/models/index.php.

## Introduction

Since 1876, centrioles and centrosomes were shown to be involved in organizing fibrillar structures, including the spindle and mitotic asters within the cell as well as cilia and flagella in cells of many tissues [1]. The centrosome is a remarkable molecular machine capable of self-replication, whose core is constituted by two centrioles [2], highly structured macromolecular complexes typically consisting of nine microtubule-triplet-blades arranged in a cylinder, which also form the basal bodies required for the formation of cilia and flagella. Centrioles were probably present in the common ancestor of all eukaryotes [3] but, contrary to what was previously thought, they are not required for general mitosis, cell migration, and axonal growth [4]. Instead, these processes require pericentriolar material (PCM), a protein matrix that is the main constituent of the centrosome and apparently lacks higher order structure. Mutations in the centrosomes are related with several human diseases, most notably cancer [5] and abnormal brain development [6], [7], [8].

Recently, large-scale proteomic experiments have identified proteins localized in the human [9], [10] and the fly centrosome [11]. Motivated by this study, Nogales-Cadenas et al. [12] retrieved from public databases, such as the Human Protein Reference Database (HPRD) [13], MiCroKit [14], Gene Ontology [15] and Ensembl [16] a large number of genes annotated as centrosomal from previous literature evidence as well as human orthologs of mouse centrosomal genes. A total of 465 likely centrosomal human genes, together with a rich set of biological annotations and derived information, were organized into a centralized resource named CentrosomeDB (http://centrosome.cnb.csic.es/).

The Centrosome 3D consortium is committed to analyze from a multidisciplinary point of view, including structural, cellular and computational approaches, the physiology of this organelle. From the computational side, using bioinformatics predictions, we have observed that proteins forming the centrosome tend to be longer, more widely phosphorylated, and to contain a larger fraction of disordered [17] and coiled-coil [18] residues than control proteins of the same organism [19]. In particular, regions that are predicted to be simultaneously disordered and coiled-coil constitute a signature of centrosomal proteins. We have found that intrinsically disordered regions increased during the evolution of the centrosome through large insertions. Interestingly, insertions of disordered regions occurred at a faster rate along branches of the animal tree where the number of cell types of the organism experienced a large increase [19]. This observation suggests an intriguing relationship between the molecular complexity of the centrosome and the cellular complexity of the organism. As part of the effort of the Centrosome 3D consortium, we report here large-scale homology modeling and expression assays of the human proteins whose centrosomal localization has been experimentally demonstrated.

## Methods

### Selection of the Data Set

From the 465 genes in the CentrosomeDB database [12], we selected those genes whose evidence is based either in the Andersen *et al.* proteomic experiment with the human centrosome [9], or in the manually curated HPRD database [13], or in literature evidence obtained by text mining and manually verified, or it is supported by orthology with respect to experiments with the mouse centrosome [12] or the proteomic experiment with Drosophila [11]. We obtained 361 genes with solid evidence of centrosomal localization, which are listed in Table S1, discarding 104 genes from the Centrosome DB that do not fulfill the above criteria. We considered the longest isoform of each gene, whose sequences are reported in Table S2. 500 control human genes and the 1202 isoforms associated to them were randomly extracted from the Ensembl database. Their Ensembl codes and sequences are reported in Table S3.

### Disorder, Coiled-coil and Secondary Structure Predictions

Disorder predictions were obtained with the DISOPRED2 [20], FoldIndex [21], IUPred [22] and DisEMBL [23] programs. In previous work, we tested that the first three algorithms have a large overlap with each other and produce qualitatively equivalent results. We present here predictions obtained with DISOPRED2, which was evaluated as the best among these predictors [24]. Predictions of coiled-coil residues were obtained with the NCOIL [25] and PCOILS [26] programs. Again, results are qualitatively equivalent and we show those obtained with NCOIL. Secondary structure was predicted with the PSIPRED program [27] and assigned with DSSP [28].

### Template Selection

Suitable templates were obtained using Hidden Markov Models (HMM) [29] as implemented in the HHblits tool kit downloaded from http://toolkit.genzentrum.lmu.de/hhblits/. Namely, we used the HHblits software [30] to construct HMMs for the 28,020 representative protein chains in the PDB [31] clustered at 70 percent sequence identity. HHblits uses secondary structure to improve the constructed HMM. We searched each query sequence against these HMMs, keeping only highly significant matches (i.e. probability of being a true positive higher than 95%) with more than 30 residues and more than 20% sequence identity, which is considered as the minimum identity for obtaining reliable structural models. In order to retrieve structures with high sequence identity that are not chosen as representative structures to construct the HMMs, we searched with BLASTP [32] all 45,543 protein chains in the PDB clustered at 100 percent sequence identity, keeping only matches above 70% sequence identity. For each query sequence, we selected the templates yielding the maximum number of identical residues. Overlapping templates were kept if the less favored template contributes at least 30 new residues to be modeled.

### Homology Modeling

For proteins with more than 20% and less than 95% sequence identity with a template structure in the PDB, structural models were built from the query-template alignment using the MODELLER program [33]. Model quality was assessed with the empirical energy function DOPE, implemented in MODELLER [34], with an empirical folding free energy function based on contact interactions [35] and with the program ProCheck [36], which checks the stereochemical quality of a protein structure, analyzing its overall and residue-by-residue geometry. Models were refined in order to avoid atomic clashes, allowing small relaxation through the following protocol: (1) System preparation: Hydrogen atoms and protons were added to the protein molecule using the program PDB2PQR [37] with the AMBER10 force-field [38] at pH 6.5; A water box of 10Å thickness was built around the protein with the program TLEAP [39] using the TIP3P model of water molecules [40]. Cl- and Na+ ions were added to neutralize when necessary. (2) Relaxation: The structure was refined by applying energy minimization followed by heating to 298 K, equilibration and cooling. No molecular dynamics per se was carried out due to the fact that, in many cases, models are quite small or too fragmented to be stable on their own. We employed NAMD 2.8 (Nano-scale Molecular Dynamics) [41] with the AMBER10 force field for the protein and the TIP3P model for water. First, the energy of the water molecules and ions was minimized keeping the protein fraction fixed. Second, the whole system was equilibrated at a constant temperature of 298 K, slowly reducing the constraints on the protein structure. Finally, the system was cooled, reducing the temperature from 298 K to 273 K with decrements of 1 K.

### Cloning Centrosomal Genes

Cloning facilities at the CNIO in Madrid, at the IBMB and the CRG in Barcelona, and the company GenCust, produced clones of 138 centrosomal genes (Table S4), which are available upon request for academic use. Centrosomal proteins were cloned into pOPIN vectors using the In-Fusion™ PCR cloning method, a versatile ligation-independent cloning system engineered for high throughput screening [42]. Three different pOPIN vectors were used in this study: pOPINJ, pOPINS and pOPINM, utilizing the cleavable fusion tags His-GST, His SUMO and His-MBP, respectively. These vectors facilitate the expression of the cloned gene in *E. coli* or human cells and they can also be used to generate baculoviruses for insect cell infection.

### Selection of Putative Globular Domains for Experimental Study

Putative globular domains were predicted for 208 selected centrosomal proteins of particular experimental interest by combining domain predictions through the SMART web server [43], disorder predictions [20], coiled-coil predictions [25] and sequence alignments of query centrosomal proteins against representative protein sequences in the PDB clustered at 50% sequence identity. Sequences were parsed into predicted SMART domains, which were given higher priority, coiled-coil stretches (consecutive stretches of more than 20 predicted coiled-coil residues) and disordered stretches (consecutive stretches of more than 20 predicted disordered residues). Regions longer than 40 residues that were not classified as none of the above were aligned against representative sequences in the PDB using the program SABERTOOTH [44], which performs accurate alignments between distant homologs by aligning predicted structural profiles, is minimally influenced by sequence identity, and measures the significance of the alignment through a Z score.

Putative globular domains were identified if one of these conditions holds: (1) SMART finds a significant match with a known protein family over at least 30 residues; (2) SABERTOOTH finds a significant match with Z score >3 with a protein in the PDB over more than 40 residues, and the sum of disordered and coiled-coil residues is below 30%. Domains with more than 40% sequence identity with structures in the PDB were considered of little interest and discarded from further experimental study. We finally obtained 173 putative globular domains from which we selected for experimental studies 65 constructs belonging to 50 proteins (Table S5). Note that these putative domains selected for experimental study do not necessarily coincide with the structural domains predicted through our homology modeling procedure, since in this case we also used information about disorder, coiled-coil, and functional domains.

### Expression of Full-length Proteins and Predicted Globular Domains

Full-length proteins corresponding to the longest isoform of centrosomal genes and selected globular domains were expressed and purified in three different labs (CNIO, IBMB and CRG), with common standardized protocols. For the expression tests the recombinant plasmids were used to transform *E. coli* B834(DE3) and Rosetta(DE3) pLysS cells. Cells were grown in LB media (2 ml) with appropriate antibiotics and induced at an OD600 of 0.8 with 0.3 mM IPTG. Two different temperatures were tested, harvesting cells after 3 h at 37°C and after 20 h at 20°C. In parallel, cells were also grown in auto-induction media and harvested after 20 h at 20°C, with shaking at 210rpm. Cells were lysed in standard buffers (50 m*M* Tris pH 8.5, 400 m*M* NaCl, 0.05% (*v/v*) Tween20) and overexpressed proteins purified either using NTA Ni spin columns or paramagnetic beads. Expression and solubility of the full-length proteins and the domains were checked by SDS-PAGE or Western Blot technique. The soluble proteins were confirmed by MALDI-MS analysis. Selected targets were also tested using baculovirus expression system.

### Antibody Production

The functional facility of the consortium at the CRG produced 44 antibodies against 40 centrosomal proteins. Furthermore, 38 antibodies against 27 centrosomal proteins were produced by the company Eurogentech. These available antibodies are listed in Table S6. We also report in Table S7 antibodies against centrosomal proteins that were commercially available prior to our study.

## Results

### Predicted Disordered and Coiled-coil Fragments

More than 57% of the residues in human centrosomal proteins are predicted to be disordered in the native state. This fraction is significantly larger than for control human proteins, for which the fraction of predicted disordered residues, obtained with the same method as for centrosomal proteins, is 39%. These results hold for the longest isoforms. 72% of the centrosomal genes and 56% of the control genes have more than one isoform. In both cases, for these genes the shortest isoform is less disordered and less coiled-coil than the longest one, although this difference is only marginally significant (for instance, the disorder content is respectively 37.8% and 40.5% for the shortest and longest isoform of control genes, with a statistical error of 1%).

**Figure 1:**
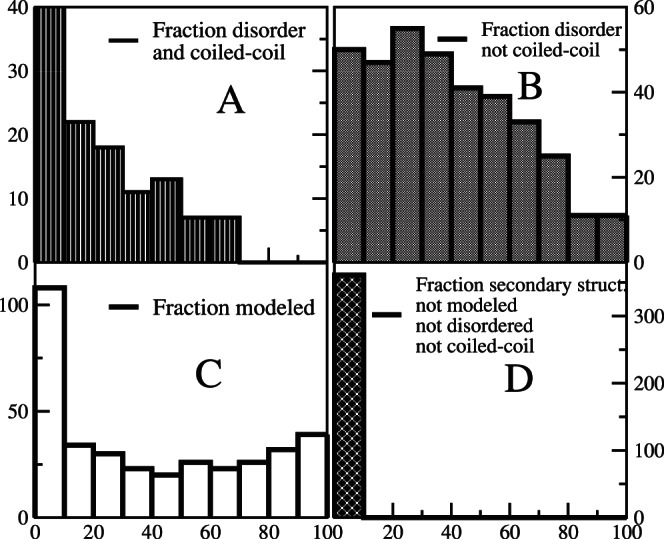
Fraction of protein length that is predicted to be disordered, coiled-coil, or modeled by homology. The plots represent the distribution of the percentage of protein length that has been (A) predicted to be disordered and coiled-coil at the same time; (B) Predicted to be disordered and not coiled-coil; (C) modeled; (D) Predicted to have regular secondary structure and not to be disordered neither coiled-coil, but not modeled.

The distribution of the fraction of the longest isoform that is predicted to be disordered is shown in Fig. 1, distinguishing regions predicted to be disordered and coiled-coil and disordered and not coiled-coil. There is a significant positive propensity to predict a residue as coiled-coil if it is predicted to be disordered: 12.4% of the residues are predicted to be disordered and coiled-coil at the same time, compared with only 0.7% that are predicted to be coiled-coil but not disordered. Therefore, the propensity is *Prop*(coil&disorder) = log(*P*(coil&disorder)-log(*P*(coil))-lof(*P*(disorder)) = 0.51. 55 proteins (15%) contain disordered and coiled-coil fragments that cover more than 20% of their length. The distribution of the fraction of the protein that is predicted as disordered and coiled-coil is shown in Fig. 1A. The same distribution for regions predicted to be disordered and not coiled-coil is shown in Fig. 1B. For control human proteins the fraction of residues predicted to be coiled-coil and disordered is much smaller (3.3%), and only 0.7% of the residues are predicted as coiled-coil and not disordered, resulting in a positive propensity between coiled-coil and disorder, Prop(coil&disorder) = 0.75.

These residues predicted to be both disordered and coiled-coil may represent disordered regions that lack stable structure unless they interact with a binding partner, and take coiled-coil structure only upon binding. The fact that coiled-coil proteins can be disordered has been shown for several proteins, for example in the case of the Myc protein interacting with a competitor of its natural partner [45], and it is consistent with the finding that the sequence complexity of coiled-coil proteins is typically lower than for globular proteins [46].

### Homology Modeling

We modeled or retrieved the structures of 384 domains contained in 277 proteins. For 84 proteins (23%), no suitable templates were found. For these proteins, on the average 74% of the residues are predicted to be disordered in their native state and only 19 proteins are predicted to possess secondary structure in more than 30% of their residues. Globally, 27.6% of the residues were modeled. The histogram of the fraction of protein length that is modeled is shown in Fig. 1C, where one can see that, for most proteins, less than 50% of the length could be modeled. This lack of models is mainly due to structural disorder: 76% of the residues that were not modeled were predicted to be either disordered or coiled-coil, and the fraction of protein for which we could not build structural models, and which we predicted to possess secondary structure and to be neither disordered nor coiled-coil, was at most 10% (see Fig. 1D).

**Figure 2:**
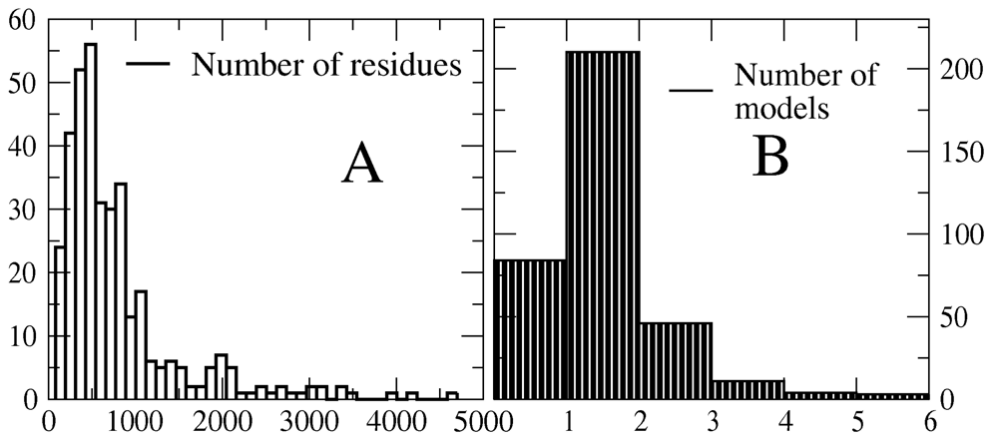
Number of residues and number of models for each protein. The plots represent the distribution of the number of residues of the longest isoform of centrosomal genes (A) and the number of structural models obtained for each protein (B).

**Figure 3:**
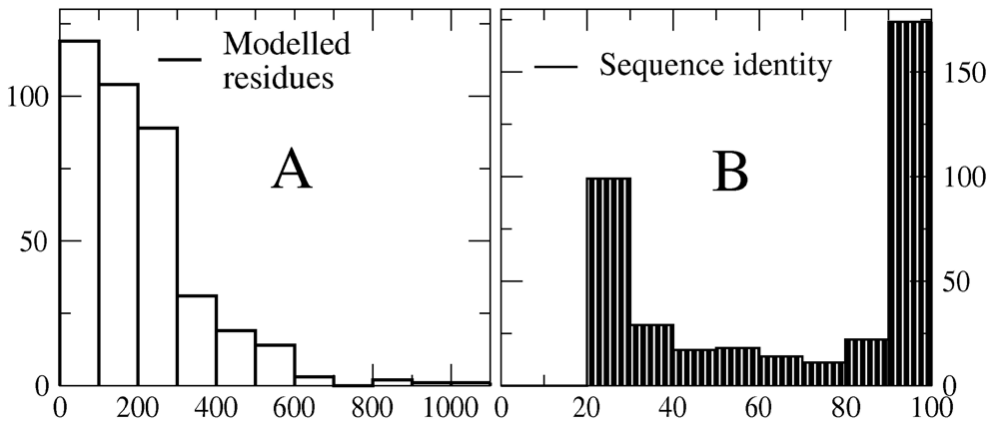
Summary of the structural models, either built by homology or retrieved from the PDB. The plots represent the distribution of the length (A) and sequence identity between query and template protein (B) for the 362 modeled fragments.

The length distribution of the 361 proteins (longest isoform of each centrosomal gene) is represented in Fig. 2A, where one can see that some proteins are extremely long. By contrast, the number of structural models built for each protein is almost in all cases smaller than 5 (Fig. 2B). The mean length of modeled fragments is 211 residues, ranging from 31 to 2922 residues (Fig. 3A). The distribution of sequence identity, plotted in Fig. 3B, is bimodal, with peaks at low and high identity: 99 fragments have less than 30% identity and 174 fragments have more than 90% identity with their PDB template. These templates with more than 95% identity were downloaded from the PDB, whereas lower identity templates were subject to the modeling procedure described in Methods.

**Figure 4:**
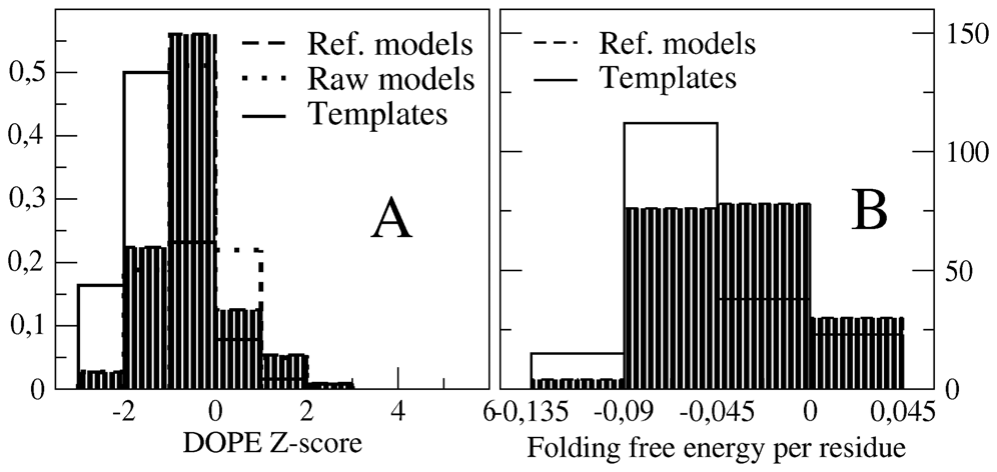
Empirical energy functions evaluated for each models and for the corresponding region of the template show that the predicted stability decrease is moderate.

The maximum length of gap regions modeled without a template was of 6 residues or fewer. Longer gaps cannot be reliably built, and they were left as unresolved structure. The DOPE energy, normalized so to transform it into a Z-score, is only slightly higher for the modeled sequence than for the template sequence (see Fig. 4A). Moderate energy structures could be slightly improved through the refinement protocol, but no improvement was achieved for high energy models, which may correspond to incorrect alignments. Similar results were obtained with the folding free energy function of Ref. [35], see Fig. 4B. Model quality was also assessed with ProCheck, which shows that the fraction of residues in disallowed regions of the Ramachandran plot increases not more than 4% from the template to the model, and that the number of residue pairs closer than 2.6Å (bad contacts) is on the average the same in the templates and the models. Based on these results, we conclude that the quality of templates and models is similar enough. However, some models had to be discarded by visual inspection, because they were very fragmented or presented too little secondary structure.

### Secondary Structure

**Figure 5:**
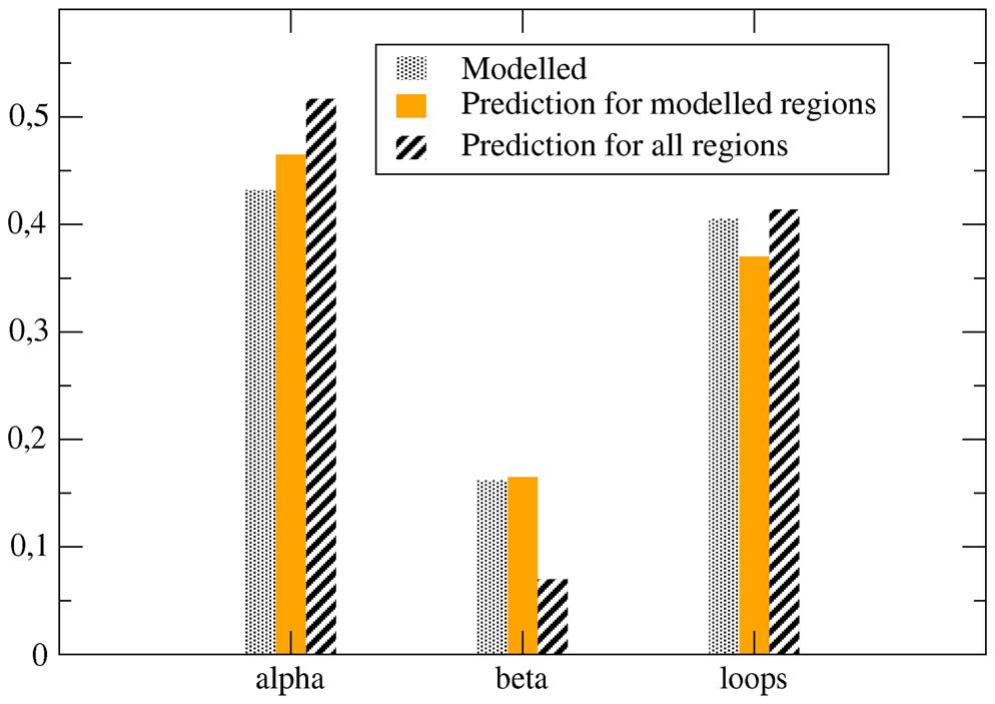
Frequency of the three main secondary structure classes for modeled residues (gray: DSSP of the template; yellow: PSIPRED prediction) and for all residues (pink). One can see that the set of all residues is strongly diminished in beta structures.

Helices prevail over strands in modeled regions (44.8% against 16.8% as assigned by the program DSSP, which identifies secondary structures based on structural information, and 46.3 against 16.6% as predicted by the program PSIPRED, which only uses sequence information), and even more in the whole centrosomal proteome (52.0% against 7.0%, as predicted by PSIPRED), as expected due to the high incidence of coiled-coils and disordered loops. The frequency of predicted secondary structure classes is shown in Fig. 5.

For control human proteins, 34.5% of the residues are predicted as helical and 12.8% are predicted as strands, which means that centrosomal proteins are enriched in helical structures and depleted in beta strands. This difference between centrosomal and control proteins is mainly due to the large fraction of centrosomal residues predicted to be at the same time disordered and alpha-helical. In fact, of the residues predicted to be disordered in centrosomal proteins, 53% are predicted to be helical by PSIPRED and only 47% are predicted to be loops. In contrast, the fraction of predicted disordered residues of control human proteins that are predicted to be helical is only 25%, and the fraction predicted to be loop is 73%. As a consequence, 30.0% of the residues in centrosomal proteins are predicted to be disordered and helical, whereas this fraction is 9.7% in control proteins. This excess of residues predicted to be disordered and helical (20.3%) accounts for the difference between centrosomal and control proteins regarding helical residues (17.5%) and disordered residues (19.5%).

### Online Database

We stored at the web site http://ub.cbm.uam.es/centrosome/models/index.php a database containing homology models and disorder and coiled-coil predictions for 361 human centrosomal proteins. For each protein there are available for online visualization and download disorder, coiled-coil and secondary structure predictions, homology models, and links to the UniProt (www.uniprot.org) and Centrosomedb [12] page. The number of modeled regions of each protein is indicated in parenthesis in the summary page, and for each model the user can download or visualize the three-dimensional structure superimposed with the template, the sequence alignment with the template, and the DOPE energy profile that identifies high energy regions that may be not well modeled. Models are linked to the PDB page of the template. The full set of models (only structures with less than 95% sequence identity) can be downloaded from the url http://ub.cbm.uam.es/centrosome/models/models_coordinates_95.tgz.

### Modularity

**Figure 6:**
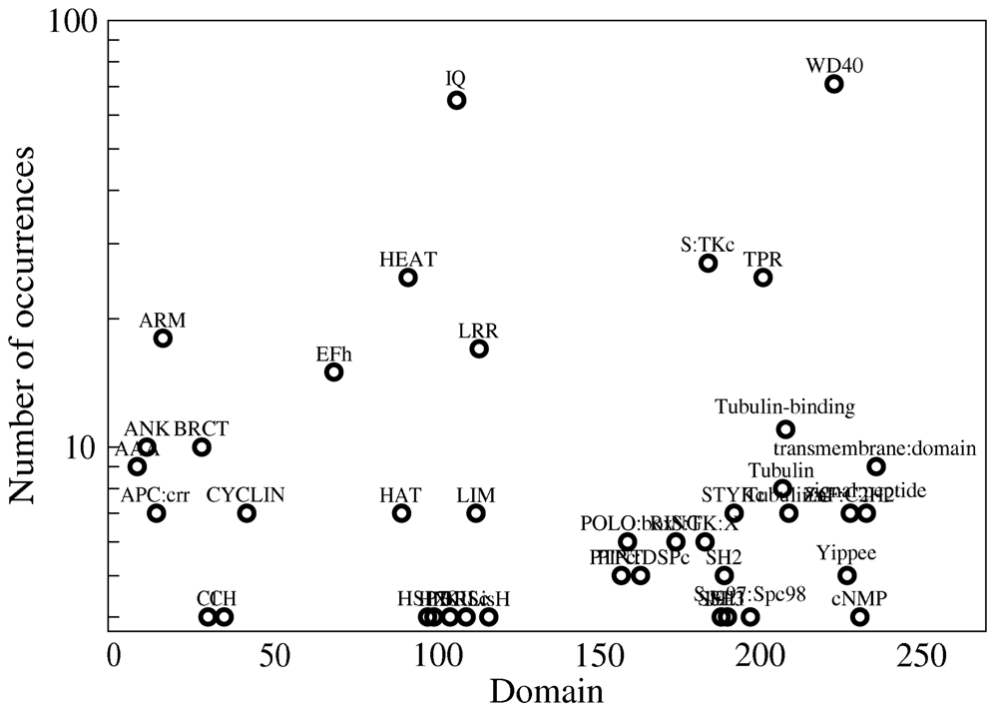
Number of occurrences of domains predicted by SMART. Only domains with more than 3 occurrences are shown.

Centrosomal proteins are highly modular. Besides coiled-coil regions, by far the most common structural motif, SMART identifies 719 evolutionary domains that belong to 239 types. The most frequent domains are the WD40 domain (71 occurrences), the IQ domain (65), the Serine Threonine Kinase domain (27), the TPR and HEAT domains (25), the ARM (18), LRR (17), EFh (15) and Tubuline binding (11) motifs. The number of occurrences for each domain in centrosomal proteins is represented in Fig. 6. Several modeled regions are constituted by multiple identical domains.

### Protein-protein Interactions

**Figure 7:**
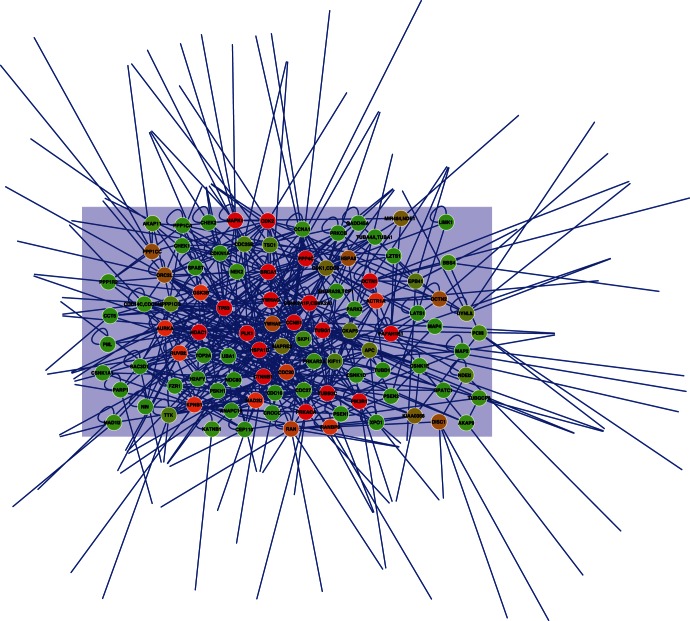
Protein-protein interaction networks for interactions experimentally observed for human centrosomal proteins. The color code represents betweeness centrality, a graph theoretic measure of the centrality of a node in a network, red representing the most central node.

We retrieved from the public databases DIP [47], MINT [48], INTACT [49], HPRD [50] and BOIGRID [51] the experimentally known protein-protein interactions of proteins in the human centrosome. We found 354 known interactions involving 167 of the 361 proteins. The average degree is 3.65 and the clustering coefficient is only 0.10. This low clustering coefficient suggests that the network is incomplete. We represent the network with the Cytoscape software [52] and we plot it in Fig. 7, representing in color code the betweeness centrality of each protein, a graph-theoretical measure of how central in the network is a node, which measures the number of shortest paths connecting any pair of nodes that pass through the given node [53]. Below, we list the most central proteins. We indicate in the brackets after the protein name the number of interaction partners and the fraction of the protein that is predicted to be disordered: TP53 (27, 46.5%), BRCA1 (20, 71.3%), YWHAG (13, 14.6%), APC (13, 84%), TUBG1 (11, 2.4%), DCTN1 (10, 94.5%), PIK3R1 (10, 30.6%), PLK1 (8, 31.4%), PAFAH1B1 (7, 5.2%). One can see that the mean disorder of central proteins is not very different from the average disorder of centrosomal proteins, but some of the central proteins, such as DCTN1, APC and BRCA1, are extremely disordered.

### Full-length Proteins Expression and Purification

The length distribution of the 361 longest isoform of each centrosomal gene, represented in Fig. 2A, shows that the most populated length bin is for proteins slightly shorter than 500 residues, and a long tail of proteins longer than 1000 residues is present. The average length is 796±41 residues, which is significantly longer than the average length of 599±31 residues for control human proteins. One of us and coworkers observed in a previous work [19] that this difference is due to the fact that exons in human centrosomal genes are more numerous (20.3 versus 14.6) than exons in control human genes.

Out of the total number of 138 available clones of centrosomal genes (Table S4), 120 were successfully cloned in pOPIN expression vectors. Of these 120 full-length clones, 71 were subjected to high-throughput expression and purification methods, resulting in 24 soluble proteins (34%, Table S4), and 5 full-length proteins with unknown structure were expressed and purified to continue with further structural studies. The remaining 49 full-length clones were expressed and purified following a medium-low throughput method. 34 of these clones showed over-expression but only 15 were soluble and subjected to small-scale purification (31%, Table S4). Combining the two methods, only 39 out of 120 full-length proteins (32.5%) were soluble or partly soluble, in the sense that we found them both in the soluble fraction and in the pellet fraction.

Overall, centrosomal proteins were extremely tricky to handle and often the over-expression and solubility were highly sensitive to many external factors including the growth media, *E.coli* expression strains, the temperature and the induction time. Some proteins were soluble 3 hours after induction, however, by decreasing the temperature and increasing the induction time of the same culture, they showed increased over-expression and became insoluble. Furthermore, solubility was compromised in several cases once the tag was removed indicating possible folding problems.

### Domain Expression and Purification

Of the 173 domain constructs designed on the basis of the bioinformatics analysis and having unknown structure (see Methods and above), 44 domains were cloned in pOPINJ vectors and 22 in pOPINF and pOPINS vectors. All of them were expressed in high throughput conditions. Of these, only 14 (21%) were found to be soluble under the conditions tested and 5 were purified at high enough concentration for crystallization screenings (Table S5). Six additional domains were expressed in low-throughput conditions in CNIO, and 5 of them were found to be soluble and 4 were purified (see Table S5). Overall, we cloned 72 domains and found that 19 of them (26%) were soluble and 9 could be purified.

## Discussion and Conclusions

Proteins in the centrosome tend to be long, modular, disordered and coiled-coil, significantly more than control proteins of the same organism. They are formed by a large number of exons, mostly corresponding to disordered regions, coiled-coils, or short domains such as the WD40 repeat, the IQ repeat and the HEAT repeat.

Centrosomal proteins are difficult to express: only 39 of the 120 full-length proteins in our expression trials were soluble (32.5%). Isoform length and disorder content can impact solubility. We took into account the disorder and coiled-coil content for predicting putative globular domains through a bioinformatics analysis. We cloned and expressed these putative domains, but we obtained a similarly low success rate: only 19 out of 72 cloned constructs resulted in soluble proteins (26%). However, when domain prediction was coupled with low-throughput expression, the success rate greatly increased: 5 out of 6 domains cloned in these conditions were found to be soluble.

Experimental structures in the PDB or the structural models that we built through homology cover 27.6% of the length of centrosomal proteins. These modeled regions distribute quite unevenly in the predicted ordered and disordered regions. In regions predicted to be neither disordered nor coiled-coil, which represent 42.2% of centrosomal proteins, we could model 57.2% of the residues, whereas in regions predicted to be disordered or coiled-coil we could model only 5.4% of the residues. For 17.7% of the residues predicted to be in globular regions we could not find any suitable template, which demands further structural studies of globular domains in centrosomal proteins.

The main characteristics of centrosomal proteins are the numerous disorder and coiled-coil regions that make them extremely flexible and able to interact with many partners, forming intertwined coiled-coils. Interestingly, centrosomal proteins contain 30% of residues that are predicted to be disordered by DISOPRED and helical by PSIPRED, whereas this fraction is only 9.4% in control proteins. This difference accounts for most of the difference between centrosomal and control proteins concerning disordered residues (57.2% against 39%) and helical residues (52.0% against 34.5%). These regions are reminiscent of the proposed alpha-helix forming molecular recognition features (α-MoRFs), structural elements that mediate the binding events of initially disordered elements [54]. The role of intrinsically disordered protein regions in the interactions of centrosomal proteins has been experimentally demonstrated in a few cases. For instance, one of us and coworkers recently studied the N-terminal domain of the centrosomal protein TBCC that is involved in bipolar spindle formation. The TBCC-Nterm adopts a spectrin-like fold topology, and remarkably its 30-residue N-terminal fragment remains flexible and completely disordered in solution. The interaction of TBCC-Nterm with tubulin involves this unstructured region, which has been suggested to acquire structure upon interaction [55].

This structural complexity of centrosomal proteins and protein-protein interactions suggests that the centrosome will remain an important subject of structural investigation, which will probably require new experimental techniques.

## Supporting Information

Table S1 List of the 361 genes with solid evidence of centrosomal localization considered in this study.
(DOC)

Table S2 Sequence of the longest isoform of each centrosomal gene.
(FASTA)

Table S3 Sequences of the 1202 isoforms associated to 500 control human genes that were randomly extracted from the Ensembl database.
(FASTA)

Table S4 List of the clones of 138 full-length centrosomal genes produced in this study, and their experimental characterization.
(DOC)

Table S5 Domain constructs selected for experimental studies and their characterization.
(DOC)

Table S6 Antibodies against centrosomal proteins produced in this study.
(XLS)

Table S7 Commercially available antibodies against centrosomal proteins retrieved through bioinformatics analysis.
(XLS)
