# Supplementary material for: Structure and Non-Structure of Centrosomal Proteins
Source: PLoS One. 2013 May 9;8(5):e62633. doi: 10.1371/journal.pone.0062633 (PMC3650010; doi:10.1371/journal.pone.0062633)
Supplement: Table S1 — List of the 361 genes with solid evidence of centrosomal localization considered in this study. (DOC) [file pone.0062633.s001.doc]

Ensembl Gene ID Associated Gene Name

ENSG00000089336 AC002115.6

ENSG00000100181 AC005301.5

ENSG00000103540 AC012621.2

ENSG00000138107 ACTR1A

ENSG00000023516 AKAP11

ENSG00000127914 AKAP9

ENSG00000106948 AKNA

ENSG00000100931 AL136419.6

ENSG00000116127 ALMS1

ENSG00000107890 ANKRD26

ENSG00000133302 ANKRD32

ENSG00000134982 APC

ENSG00000142192 APP

ENSG00000066279 ASPM

ENSG00000149311 ATM

ENSG00000175054 ATR

ENSG00000164053 ATRIP

ENSG00000087586 AURKA

ENSG00000178999 AURKB

ENSG00000105146 AURKC

ENSG00000103126 AXIN1

ENSG00000168646 AXIN2

ENSG00000141577 AZI1

ENSG00000155096 AZIN1

ENSG00000140463 BBS4

ENSG00000089685 BIRC5

ENSG00000169594 BNC1

ENSG00000012048 BRCA1

ENSG00000102760 C13orf15

ENSG00000100629 C14orf145

ENSG00000143933 CALM2

ENSG00000070808 CAMK2A

ENSG00000168491 CCDC110

ENSG00000121289 CCDC123

ENSG00000149548 CCDC15

ENSG00000104983 CCDC61

ENSG00000175602 CCDC85B

ENSG00000133101 CCNA1

ENSG00000145386 CCNA2

ENSG00000134057 CCNB1

ENSG00000138764 CCNG2

ENSG00000112486 CCR6

ENSG00000150753 CCT5

ENSG00000079335 CDC14A

ENSG00000081377 CDC14B

ENSG00000130177 CDC16

ENSG00000117399 CDC20

ENSG00000101224 CDC25B

ENSG00000158402 CDC25C

ENSG00000004897 CDC27

ENSG00000107736 CDH23

ENSG00000170312 CDK1

ENSG00000123374 CDK2

ENSG00000136861 CDK5RAP2

ENSG00000124762 CDKN1A

ENSG00000138778 CENPE

ENSG00000117724 CENPF

ENSG00000153044 CENPH

ENSG00000151849 CENPJ

ENSG00000119397 CEP110

ENSG00000168944 CEP120

ENSG00000174799 CEP135

ENSG00000103995 CEP152

ENSG00000110274 CEP164

ENSG00000143702 CEP170

ENSG00000101639 CEP192

ENSG00000126001 CEP250

ENSG00000198707 CEP290

ENSG00000135837 CEP350

ENSG00000138180 CEP55

ENSG00000166037 CEP57

ENSG00000182923 CEP63

ENSG00000011523 CEP68

ENSG00000114107 CEP70

ENSG00000112877 CEP72

ENSG00000101624 CEP76

ENSG00000148019 CEP78

ENSG00000182504 CEP97

ENSG00000177143 CETN1

ENSG00000147400 CETN2

ENSG00000153140 CETN3

ENSG00000170004 CHD3

ENSG00000111642 CHD4

ENSG00000149554 CHEK1

ENSG00000183765 CHEK2

ENSG00000101421 CHMP4B

ENSG00000175216 CKAP5

ENSG00000074054 CLASP1

ENSG00000163539 CLASP2

ENSG00000169504 CLIC4

ENSG00000044459 CNTLN

ENSG00000170037 CNTROB

ENSG00000093010 COMT

ENSG00000166200 COPS2

ENSG00000141030 COPS3

ENSG00000138663 COPS4

ENSG00000121022 COPS5

ENSG00000168090 COPS6

ENSG00000111652 COPS7A

ENSG00000144524 COPS7B

ENSG00000198612 COPS8

ENSG00000072832 CRMP1

ENSG00000058453 CROCC

ENSG00000113712 CSNK1A1

ENSG00000141551 CSNK1D

ENSG00000213923 CSNK1E

ENSG00000101266 CSNK2A1

ENSG00000070770 CSNK2A2

ENSG00000126890 CTAG2

ENSG00000168036 CTNNB1

ENSG00000136848 DAB2IP

ENSG00000118655 DCLRE1B

ENSG00000204843 DCTN1

ENSG00000175203 DCTN2

ENSG00000137100 DCTN3

ENSG00000132912 DCTN4

ENSG00000135829 DHX9

ENSG00000162946 DISC1

ENSG00000126787 DLGAP5

ENSG00000079805 DNM2

ENSG00000143476 DTL

ENSG00000197102 DYNC1H1

ENSG00000077380 DYNC1I2

ENSG00000144635 DYNC1LI1

ENSG00000088986 DYNLL1

ENSG00000038358 EDC4

ENSG00000102119 EMD

ENSG00000159023 EPB41

ENSG00000135476 ESPL1

ENSG00000149557 FEZ1

ENSG00000213066 FGFR1OP

ENSG00000105255 FSD1

ENSG00000105325 FZR1

ENSG00000116717 GADD45A

ENSG00000057608 GDI2

ENSG00000196329 GIMAP5

ENSG00000149124 GLYAT

ENSG00000112312 GMNN

ENSG00000127955 GNAI1

ENSG00000114353 GNAI2

ENSG00000065135 GNAI3

ENSG00000090615 GOLGA3

ENSG00000144674 GOLGA4

ENSG00000082701 GSK3B

ENSG00000188486 H2AFX

ENSG00000113648 H2AFY

ENSG00000152240 HAUS1

ENSG00000137814 HAUS2

ENSG00000092036 HAUS4

ENSG00000147874 HAUS6

ENSG00000131351 HAUS8

ENSG00000113070 HBEGF

ENSG00000134248 HBXIP

ENSG00000116478 HDAC1

ENSG00000072571 HMMR

ENSG00000095066 HOOK2

ENSG00000080824 HSP90AA1

ENSG00000204388 HSPA1B

ENSG00000126803 HSPA2

ENSG00000109971 HSPA8

ENSG00000113013 HSPA9

ENSG00000125968 ID1

ENSG00000109083 IFT20

ENSG00000032742 IFT88

ENSG00000166333 ILK

ENSG00000149503 INCENP

ENSG00000129474 JUB

ENSG00000186625 KATNA1

ENSG00000140854 KATNB1

ENSG00000136813 KIAA0368

ENSG00000100578 KIAA0586

ENSG00000101004 KIAA0980

ENSG00000166004 KIAA1731

ENSG00000138160 KIF11

ENSG00000163808 KIF15

ENSG00000068796 KIF2A

ENSG00000080986 KNTC2

ENSG00000108424 KPNB1

ENSG00000131023 LATS1

ENSG00000150457 LATS2

ENSG00000182866 LCK

ENSG00000005156 LIG3

ENSG00000106683 LIMK1

ENSG00000182541 LIMK2

ENSG00000169683 LRRC45

ENSG00000133739 LRRCC1

ENSG00000061337 LZTS1

ENSG00000107816 LZTS2

ENSG00000002822 MAD1L1

ENSG00000164109 MAD2L1

ENSG00000166963 MAP1A

ENSG00000130479 MAP1S

ENSG00000078018 MAP2

ENSG00000173327 MAP3K11

ENSG00000047849 MAP4

ENSG00000164114 MAP9

ENSG00000100030 MAPK1

ENSG00000101367 MAPRE1

ENSG00000166974 MAPRE2

ENSG00000186868 MAPT

ENSG00000155130 MARCKS

ENSG00000007047 MARK4

ENSG00000071655 MBD3

ENSG00000100297 MCM5

ENSG00000147316 MCPH1

ENSG00000187778 MCRS1

ENSG00000111554 MDM1

ENSG00000125863 MKKS

ENSG00000178053 MLF1

ENSG00000157227 MMP14

ENSG00000136286 MYO1G

ENSG00000104320 NBN

ENSG00000072864 NDE1

ENSG00000166579 NDEL1

ENSG00000182636 NDN

ENSG00000104419 NDRG1

ENSG00000139350 NEDD1

ENSG00000140398 NEIL1

ENSG00000117650 NEK2

ENSG00000151414 NEK7

ENSG00000160602 NEK8

ENSG00000100503 NIN

ENSG00000143156 NME7

ENSG00000181163 NPM1

ENSG00000167005 NUDT21

ENSG00000143228 NUF2

ENSG00000137497 NUMA1

ENSG00000125450 NUP85

ENSG00000104904 OAZ1

ENSG00000136811 ODF2

ENSG00000046651 OFD1

ENSG00000085840 ORC1

ENSG00000115942 ORC2

ENSG00000007168 PAFAH1B1

ENSG00000214982 PARG

ENSG00000185345 PARK2

ENSG00000143799 PARP1

ENSG00000041880 PARP3

ENSG00000180628 PCGF5

ENSG00000078674 PCM1

ENSG00000160299 PCNT

ENSG00000178104 PDE4DIP

ENSG00000089220 PEBP1

ENSG00000083535 PIBF1

ENSG00000145675 PIK3R1

ENSG00000105647 PIK3R2

ENSG00000127445 PIN1

ENSG00000166851 PLK1

ENSG00000145632 PLK2

ENSG00000173846 PLK3

ENSG00000142731 PLK4

ENSG00000140464 PML

ENSG00000164087 POC1A

ENSG00000139323 POC1B

ENSG00000152359 POC5

ENSG00000130997 POLN

ENSG00000186951 PPARA

ENSG00000172531 PPP1CA

ENSG00000213639 PPP1CB

ENSG00000186298 PPP1CC

ENSG00000184203 PPP1R2

ENSG00000105568 PPP2R1A

ENSG00000149923 PPP4C

ENSG00000154845 PPP4R1

ENSG00000163605 PPP4R2

ENSG00000072062 PRKACA

ENSG00000114302 PRKAR2A

ENSG00000005249 PRKAR2B

ENSG00000166501 PRKCB

ENSG00000101000 PROCR

ENSG00000080815 PSEN1

ENSG00000143801 PSEN2

ENSG00000159792 PSKH1

ENSG00000159352 PSMD4

ENSG00000112245 PTP4A1

ENSG00000158079 PTPDC1

ENSG00000011454 RABGAP1

ENSG00000132341 RAN

ENSG00000099901 RANBP1

ENSG00000010017 RANBP9

ENSG00000079337 RAPGEF3

ENSG00000068028 RASSF1

ENSG00000136653 RASSF5

ENSG00000099849 RASSF7

ENSG00000135249 RINT1

ENSG00000034677 RNF19A

ENSG00000175634 RPS6KB2

ENSG00000175792 RUVBL1

ENSG00000183207 RUVBL2

ENSG00000180739 S1PR5

ENSG00000168061 SAC3D1

ENSG00000156876 SASS6

ENSG00000151466 SCLT1

ENSG00000142186 SCYL1

ENSG00000054282 SDCCAG8

ENSG00000198089 SFI1

ENSG00000129810 SGOL1

ENSG00000165480 SKA3

ENSG00000104976 SNAPC2

ENSG00000173267 SNCG

ENSG00000076382 SPAG5

ENSG00000021574 SPAST

ENSG00000186583 SPATC1

ENSG00000133104 SPG20

ENSG00000176170 SPHK1

ENSG00000063176 SPHK2

ENSG00000163611 SPICE1

ENSG00000176101 SSNA1

ENSG00000173465 SSSCA1

ENSG00000123473 STIL

ENSG00000112079 STK38

ENSG00000101109 STK4

ENSG00000170310 STX8

ENSG00000147526 TACC1

ENSG00000138162 TACC2

ENSG00000013810 TACC3

ENSG00000141556 TBCD

ENSG00000120438 TCP1

ENSG00000125409 TEKT3

ENSG00000113272 THG1L

ENSG00000131747 TOP2A

ENSG00000141510 TP53

ENSG00000088325 TPX2

ENSG00000100815 TRIP11

ENSG00000135451 TROAP

ENSG00000165699 TSC1

ENSG00000074319 TSG101

ENSG00000106477 TSGA14

ENSG00000165533 TTC8

ENSG00000112742 TTK

ENSG00000167552 TUBA1A

ENSG00000127824 TUBA4A

ENSG00000137379 TUBB

ENSG00000188229 TUBB2C

ENSG00000104833 TUBB4

ENSG00000108423 TUBD1

ENSG00000074935 TUBE1

ENSG00000131462 TUBG1

ENSG00000130640 TUBGCP2

ENSG00000126216 TUBGCP3

ENSG00000137822 TUBGCP4

ENSG00000153575 TUBGCP5

ENSG00000128159 TUBGCP6

ENSG00000115514 TXNDC9

ENSG00000130985 UBA1

ENSG00000167671 UBXN6

ENSG00000108651 UTP6

ENSG00000126756 UXT

ENSG00000103043 VAC14

ENSG00000156787 WDR67

ENSG00000116213 WDR8

ENSG00000165392 WRN

ENSG00000082898 XPO1

ENSG00000100027 YPEL1

ENSG00000175155 YPEL2

ENSG00000090238 YPEL3

ENSG00000166793 YPEL4

ENSG00000119801 YPEL5

ENSG00000108953 YWHAE

ENSG00000170027 YWHAG

ENSG00000138311 ZNF365
