# Supplementary material for: Structure and Non-Structure of Centrosomal Proteins
Source: PLoS One. 2013 May 9;8(5):e62633. doi: 10.1371/journal.pone.0062633 (PMC3650010; doi:10.1371/journal.pone.0062633)
Supplement: Table S4 — List of the clones of 138 full-length centrosomal genes produced in this study, and their experimental characterization. (DOC) [file pone.0062633.s004.doc]

Supplementary table 3 for the paper

“Structure and non-structure of centrosomal proteins”: Full length clones.

A: Genes cloned in pOPINJ expression vectors.

| **Protein** | **Swissprot number** | **Soluble** | **Purified** | **Lab** |
| --- | --- | --- | --- | --- |
| ACTR1A | P61163 | Yes |  | CNIO |
| AURKA | O14965 | Yes |  | CNIO |
| AURKB | Q96GD4 | Yes |  | CNIO |
| BBS4 | Q96RK4 | Yes |  | CNIO |
| BIRC5 | O15392 | Yes | Yes | CNIO |
| BRCA1 | P38398 |  |  | CNIO |
| C14orf145 | Q6ZU80 |  |  | CNIO |
| C14orf94 | Q9H6D7 |  |  | CNIO |
| CAMK2A | Q9UQM7 |  |  | CNIO |
| CCDC5 | Q96CS2 | Yes |  | CNIO |
| CCNA2 | P20248 | Yes |  | CNIO |
| CCNB1 | P14635 |  |  | CNIO |
| CCT5 | P48643 | Yes | Yes | CNIO |
| CDC16 | Q13042 |  |  | CNIO |
| CDC2 | P06493 |  |  | CNIO |
| CDC20 | Q12834 | Yes |  | CNIO |
| CDC25B | P30305 |  |  | CNIO |
| CENPH | Q9H3R5 | Yes |  | CNIO |
| CENPJ* | Q9HC77 |  |  |  |
| CEP27 | Q9NVX0 |  |  | CNIO |
| CEP57 | Q86XR8 |  |  | CNIO |
| CEP70 | Q8NHQ1 | Yes |  | CNIO |
| CEP72 | Q9P209 |  |  | CNIO |
| CEP76 | Q8TAP6 |  |  | CNIO |
| CEP164* | NP_055771 |  |  |  |
| CEP170* | NP_001035864 |  |  |  |
| CETN1 | Q12798 | Yes | Yes | CNIO |
| CETN3 | O15182 | Yes | Yes | CNIO |
| CHEK2 | O96017 |  |  | CNIO |
| CNTROB | Q8N137 |  |  | CNIO |
| CSNK1D | P48730 | Yes |  | CNIO |
| CSNK1E | P49674 |  |  | CNIO |
| CTAG2 | O75638 |  |  | CNIO |
| DCTN3 | O75935 |  |  | CNIO |
| DCTN4 | Q9UJW0 |  |  | CNIO |
| DISC1* | Q9NRI5 |  |  |  |
| DLG7 | Q15398 |  |  | CNIO |
| DYNC1I2 | Q13409 |  |  | CNIO |
| DYNC1LI1 | Q9Y6G9 |  |  | CNIO |
| DYNLL1 | P63167 |  |  | CNIO |
| FEZ1 | Q99689 |  |  | CNIO |
| FGFR10P | P51684 |  |  | CNIO |
| FSD1 | NP_077309 |  |  | CNIO |
| GADD45A | P24522 | Yes | Yes | CNIO |
| GCP4 | Q9UGJ1 |  |  | CNIO |
| GDI2 | P50395 |  |  | CNIO |
| GIMAP5 | Q96F15 |  |  | CNIO |
| H2AFY | O75367 |  |  | CNIO |
| HMMR | O75330 |  |  | CNIO |
| IFT20 | Q8IY31 |  |  | IBMB |
| JUB | Q96IF1 |  |  | IBMB |
| KATNB1 | Q9BVA0 | Yes |  | IBMB |
| KIAA0841 | O94927 |  |  | IBMB |
| KIF11 | P52732 |  |  | IBMB |
| KIF2A | BC031828 | Yes |  | IBMB |
| LATS1 | O95835 |  |  | IBMB |
| LRRC45 | Q96CN5 | Yes |  | IBMB |
| LRRIQ2 | Q8IW35 |  |  | IBMB |
| MAD1L1 | Q9Y6D9 | Yes |  | IBMB |
| MAP3K11 | Q16584 |  |  | IBMB |
| MAPRE1 | Q15691 | Yes |  | IBMB |
| MAPRE2 | Q15555 |  |  | IBMB |
| MARCKS | P29966 | Yes |  | IBMB |
| MLF1 | P58340 |  |  | IBMB |
| MYO1G | NP_149043 |  |  | IBMB |
| NEDD1 | Q8NHV4 |  |  | IBMB |
| NEK2 | P51955 |  |  | IBMB |
| NDN | Q99608 | Yes |  | IBMB |
| NPM1 | P06748 |  |  | IBMB |
| NUDT21 | O43809 | Yes |  | IBMB |
| NUP85 | NP_079120 |  |  | IBMB |
| PAFAH1B1 | P43034 | Yes |  | IBMB |
| PARP3 | Q9Y6F1 |  |  | IBMB |
| PCGF5 | Q86SE9 |  |  | IBMB |
| PLK1 | P53350 |  |  | IBMB |
| PLK3 | Q9H4B4 |  |  | IBMB |
| PLK4 | O00444 |  |  | IBMB |
| PPP2R1A | P30153 | Yes |  | IBMB |
| PPP4C | P60510 | Yes |  | IBMB |
| PRKAR2A | P13861 |  |  | IBMB |
| PRKAR2B | P31323 |  |  | IBMB |
| PROCR | Q9UNN8 |  |  | IBMB |
| PSEN1 | P49768 |  |  | IBMB |
| PSKH1 | P11801 |  |  | IBMB |
| PTP4A1 | Q93096 | Yes |  | IBMB |
| RABGAP1 | NP_036329 |  |  | IBMB |
| RANBP1 | P43487 |  |  | IBMB |
| RNF19A | Q9NV58 |  |  | IBMB |
| SAC3D1 | A6NKF1 | Yes |  | IBMB |
| SASS6 | Q6UVJ0 |  |  | IBMB |
| SCLT1 | Q96NL6 | Yes |  | IBMB |
| SCYL1 | Q96KG9 |  |  | IBMB |
| SNCG | O76070 | Yes | Yes | IBMB |
| SPATC1 | NP_940974 |  |  | IBMB |
| SSNA1 | O43805 |  |  | IBMB |
| SSSCA1 | O60232 |  |  | IBMB |
| TACC3 | Q9Y6A5 |  |  | IBMB |
| TEKT3 | Q9BXF9 |  |  | IBMB |
| THG1L | NP_060342 |  |  | IBMB |
| TTC8 | Q8TAM2 |  |  | IBMB |
| TTK | P33981 |  |  | IBMB |
| TUBA1A | Q71U36 |  |  | IBMB |
| TUBA4A | P68366 | Yes |  | IBMB |
| TUBB2C | BC004188 |  |  | IBMB |
| TUBB4 | P04350 |  |  | IBMB |
| TUBD1 | Q9UJT1 |  |  | IBMB |
| TUBE1 | Q9UJT0 |  |  | IBMB |
| TUBG1 | P23258 | Yes | Yes | IBMB |
| TUBGCP2 | Q9BSJ2 | Yes |  | IBMB |
| TUBGCP3 | Q96CW5 |  |  | IBMB |
| TUBGCP5 | Q96RT8 |  |  | IBMB |
| TXNDC9 | O14530 | Yes | Yes | IBMB |
| WDR51A | Q8NBT0 |  |  | IBMB |
| WDR51B | BC026080 |  |  | IBMB |
| WDR67 | Q96DN5 |  |  | IBMB |
| WDR8 | Q9P2S5 | Yes |  | IBMB |
| YPEL1 | O60688 | Yes | Yes | IBMB |
| YPEL2 | Q96QA6 | Yes | Yes | IBMB |
| YWHAE | P62258 | Yes |  | IBMB |
| YWHAG | P61981 | Yes |  | IBMB |

B. Genes available in pUC57 vectors, which could not be cloned in expression vectors

| AURKC | **Q9UQB9** |  |  | CNIO |
| --- | --- | --- | --- | --- |
| C5orf37 | **Q8NA72** |  |  | CNIO |
| CCDC123 | **Q96ST8** |  |  | CNIO |
| CENPE | **Q02224** |  |  | CNIO |
| CEP110 | **Q7Z7A1** |  |  | CNIO |
| CEP135 | **Q66GS9** |  |  | CNIO |
| CEP192 | **Q8TEP8** |  |  | CNIO |
| CEP350 | **Q5VT06** |  |  | CNIO |
| CROCC | **Q5TZA2** |  |  | CNIO |
| GOLGA3 | **Q08378** |  |  | CNIO |
| ESPL1 | **Q14674** |  |  | IBMB |
| KIAA0368 | **Q5VYK3** |  |  | IBMB |
| KIF20B | Q96Q89 |  |  | IBMB |
| PCM1 | **Q15154** |  |  | IBMB |
| PCNT | **O95613** |  |  | IBMB |
| TRIP11 | **Q15643** |  |  | IBMB |
| TSGA14 | **Q9BYV8** |  |  | IBMB |
| TUBGCP6 | **Q96RT7** |  |  | IBMB |
