# Supplementary material for: Structure and Non-Structure of Centrosomal Proteins
Source: PLoS One. 2013 May 9;8(5):e62633. doi: 10.1371/journal.pone.0062633 (PMC3650010; doi:10.1371/journal.pone.0062633)
Supplement: Table S5 — Domain constructs selected for experimental studies and their characterization. (DOC) [file pone.0062633.s005.doc]

**Supplementary table 4 for the paper**

**“Structure and non-structure of centrosomal proteins”.**

**Domains cloned in pOPINJ vectors**

| **Protein** | **Swissprot number** | **Domain** | **Soluble** | **Purified** | **Lab** |
| --- | --- | --- | --- | --- | --- |
| JUB | Q96IF1 | 336-530 | Yes | Yes | IBMB |
| KATNB1 | Q9BVA0 | 9-301 |  |  | IBMB |
|  |  | 9-340 |  |  | IBMB |
|  |  | 421-653 |  |  | IBMB |
| LRRC45 | Q96CN5 | 29-254 | Yes |  | IBMB |
| LRRIQ2 | Q8IW35 | 15-206 |  |  | IBMB |
| MAP3K11 | Q16584 | 115-382 | Yes |  | IBMB |
| MYO1G | NP_149043 | 10-637 |  |  | IBMB |
|  |  | 10-711 |  |  | IBMB |
| NDN | Q99608 | 96-306 |  |  | IBMB |
| NUP85 | NP_079120 | 54-605 |  |  | IBMB |
|  |  | 65-636 |  |  | IBMB |
| PCGF5 | Q86SE9 | 9-83 |  |  | IBMB |
|  |  | 9-100 |  |  | IBMB |
| PLK3 | Q9H4B4 | 51-332 |  |  | IBMB |
|  |  | 51-644 |  |  | IBMB |
|  |  | 343-644 |  |  | IBMB |
|  |  | 461-644 |  |  | IBMB |
| PPP4C | P60510 | 6-287 |  |  | IBMB |
| PSKH1 | P11801 | 97-380 |  |  | IBMB |
| RABGAP1 | NP_036329 | 144-1046 |  |  | IBMB |
|  |  | 144-487 | Yes |  | IBMB |
|  |  | 536-849 | Yes | Yes | IBMB |
|  |  | 536-968 | Yes | Yes | IBMB |
|  |  | 144-849 | Yes |  | IBMB |
|  |  | 536-1046 | Yes |  | IBMB |
| SCYL1 | Q96KG9 | 29-553 |  |  | IBMB |
| TEKT3 | Q9BXF9 | 97-479 |  |  | IBMB |
| THG1L | NP_060342 | 30-291 |  |  | IBMB |
| TTC8 | Q8TAM2 | 125-529 |  |  | IBMB |
|  |  | 241-510 | Yes |  | IBMB |
| TUBE1 | Q9UJT0 | 1-455 |  |  | IBMB |
| TUBGCP3 | Q96CW5 | 247-738 |  |  | IBMB |
|  |  | 285-880 |  |  | IBMB |
| TUBGCP5 | Q96RT8 | 266-916 |  |  | IBMB |
|  |  | 266-1017 |  |  | IBMB |
| TXNDC9 | O14530 | 6-181 |  |  | IBMB |
| WDR51B | BC026080 | 9-299 |  |  | IBMB |
|  |  | 9-404 |  |  | IBMB |
| WDR67 | Q96DN5 | 2-334 |  |  | IBMB |
|  |  | 41-334 |  |  | IBMB |
|  |  | 41-404 |  |  | IBMB |
|  |  | 411-652 |  |  | IBMB |
| WDR8 | Q9P2S5 | 10-456 |  |  | IBMB |

| **Protein** | **Swissprot number** | **Domain** | **Soluble** | **Purified** | **Lab** |
| --- | --- | --- | --- | --- | --- |
|  |  |  |  |  |  |
| PLK1 | P53350 | 53-305 | yes | yes | CNIO |
| PLK2 | NP_006613.2 | 71-359 | yes | yes | CNIO |
| PLK3 | Q9H4B4 | 62-314 | yes | yes | CNIO |
|  |  |  |  |  |  |
| PLK4 | O00444 | 12-265 | yes |  | CNIO |
|  |  |  |  |  |  |
| TACC1 | BC041391 | 522-731 |  |  | CNIO |
|  |  |  |  |  |  |
| TACC3 | Q9Y6A5 | 637-838 | yes | yes | CNIO |
|  |  |  |  |  |  |

Domains in pOPINF vector and pOPINS vector

| **Protein** | **Swissprot number** | **Domain** | **Soluble** | **Purified** | **Lab** |
| --- | --- | --- | --- | --- | --- |
| AURORA B | Q96GD4 | 77-344 |  |  | CRG |
| AURORA C | Q9UQB9 | 1-306 |  |  | CRG |
| BNC1 | Q01954 | 1-266 |  |  | CRG |
| CCDC100 | Q8N960 | 1-151 | yes | yes | CRG |
|  |  | 1-114 | yes | no | CRG |
| CDC20 | Q12834 | 81-498 | yes | no | CRG |
| CEP72 | Q9P209 | 1-160 |  |  | CRG |
| DYNC1I2 | Q13409 | 110-612 |  |  | CRG |
|  |  | 272-601 |  |  | CRG |
| DYNC1LI1 | Q9Y6G9 | 1-517 | yes | no | CRG |
| EDC4 | Q6P2E9 | 155-394 |  |  | CRG |
|  |  | 165-384 |  |  | CRG |
| ESPL1 | Q14674 | 1692-2113 |  |  | CRG |
| GCP4 | Q9UGJ1 | 1-651 |  |  | CRG |
| GLYAT | Q6IB77 | 1-294 |  |  | CRG |
| KIAA0368 | J3KN16 | 683-1419 |  |  | CRG |
| LATS1 | O95835 | 698-1020 | yes | yes | CRG |
|  |  | 705-1082 |  |  | CRG |
| MPHOSPH1 | Q96Q89 | 56-495 |  |  | CRG |
| NEDD1 | Q8NHV4 | 1-330 |  |  | CRG |
| RNF19 | Q9NV58 | 130-346 |  |  | CRG |
| SAC3D1 | A6NKF1 | 72-344 |  |  | CRG |
